# Visuo-haptic prediction errors: a multimodal dataset (EEG, motion) in BIDS format indexing mismatches in haptic interaction

**DOI:** 10.3389/fnrgo.2024.1411305

**Published:** 2024-06-05

**Authors:** Lukas Gehrke, Leonie Terfurth, Sezen Akman, Klaus Gramann

**Affiliations:** Biological Psychology and Neuroergonomics, Department of Psychology and Ergonomics, Technological University Berlin, Berlin, Germany

**Keywords:** neuroergonomics, BIDS, EEG, prediction error, motion, virtual reality

## 1 Introduction

One of the key challenges in the design of immersive virtual reality (VR) is to create an experience that mimics the natural, real world as closely as possible. The overarching goal is that users “treat what they perceive as real” and consequently feel present in the virtual world (Slater, 2009). To feel present in an environment, users need to establish a dynamic and precise interaction with their surroundings. This allows users to infer the causal structures in the (virtual) world they find themselves in and develop strategies to deal with uncertainties (Knill and Pouget, 2004).

Here, we present a data set that indexes interaction realism in VR. By violating users' predictions about the VR's interaction behavior in an “oddball-like” manner (Sutton et al., 1965), labels with high temporal resolution were obtained (that describe the interaction); see our previous publications (Gehrke et al., 2019, 2022).

### 1.1 Background and related work

Today, the brain is frequently conceived of as creating a model of its environment in the constant game of predicting the causes of its available sensory data (Rao and Ballard, 1999; Friston, 2010; Clark, 2013). In this predictive coding conception, probabilistic analyzes of previous experiences drive inferences about which actions and perceptual events are causally related. This is inherently tied to the body's capacity to act on the environment, rendering the action–perception cycle of cognition into an embodied process (Friston, 2012). When all movement-related sensory data (i.e., sensorimotor data) are consistent with the predicted outcome of an action, the action is regarded as successful. However, when a discrepancy between the predicted and the actual sensorimotor data are detected, a prediction error occurs, and attention will be directed to correct for the discrepancy in real time (Savoie et al., 2018). In their work, Savoie et al. (2018) manipulated the control-to-display ratio in a quarter of the trials. In the manipulated trials, a dot moved at 45° offset compared to the real hand motion during a reach to a target. The authors found electroencephalographic data (EEG) data to reflect this prediction error in sensorimotor mapping.

Therefore, the fast and accurate detection of such discrepancies is crucial for performing precise interactions in the real as well as in virtual worlds.

The underlying mechanisms and neural foundations of predictive coding have been extensively studied; see, for example, Holroyd and Coles (2002), Bendixen et al. (2012), and Clark (2013). The frontal mismatch negativity paradigm (MMN, a type of event-related potential, also known as ERP) has often been employed to probe the predictive brain hypothesis, Stefanics et al. (2014) for a review. Lieder et al. (2013) have shown that the best-fitting explanation of MMN activity is the computation of a Bayes-optimal generative model, that is, prediction errors.

However, these research findings originate from stationary EEG protocols that require the user to passively observe presented stimuli, neglecting the embodied cognitive aspects of goal-directed behavior. As a consequence, the cortical activity patterns underlying predictive embodied processes during goal-directed movement are not fully established. How these electrocortical features reflect a perceived loss in physical immersion when interacting with virtual- and augmented reality (VR/AR) is yet to be understood.

### 1.2 A data set capturing visuo-haptic predictions in VR

The presented mobile brain/body imaging data include brain recordings via EEG and behavioral indexes, as well as motion capture during an interactive VR experience (Makeig et al., 2009; Gramann et al., 2014; Jungnickel et al., 2019). Based on the idea that the brain has evolved to optimize motor behavior by detecting sensory mismatches, we have previously leveraged these data to use the frontal “prediction error” negativity (PEN) as a feature for detection of system errors in haptic VR (Gehrke et al., 2019, 2022).

The data set is available in the Brain Imaging Data System (BIDS) format (Pernet et al., 2019; Jeung et al., 2023). In an oddball-style paradigm, haptic realism was altered, resulting in a 2 (mismatch) × 3 (level of haptic immersion) design. This allows for both, the analyzes of each main effect as well as their interaction. In the experiment, interaction realism was manipulated by adding temporally unexpected visual and haptic feedback. To this end, visuo-haptic glitches were introduced during a reaching task, similar to unexpected tones in classical auditory oddball paradigms (Sutton et al., 1965). Haptic realism was altered by adding haptic channels per condition in the experimental block design. Two haptic conditions were presented following a baseline, non-haptic, condition. Touching a surface was rendered through a vibration motor under the fingertip in one condition, and in another condition, this was further combined with rendering object rigidity (force feedback) through the use of electrical muscle stimulation (EMS).

After experiencing each haptic modality, participants rated their subjective level of presence on the Igroup Presence Questionnaire (IPQ; Schubert, 2003). This questionnaire is a scale for measuring the *subjective* sense of presence experienced in VR.

## 2 Multimodal prediction error data set

### 2.1 Participants

The experiment was approved by the local ethics committee of the Department of Psychology and Ergonomics at the TU Berlin (Ethics approval: GR1020180603). In total, 20 participants (12 female, mean age = 26.7 years, *SD* = 3.6 years) were recruited through an online tool provided by the Department of Psychology and Ergonomics of the Berlin Institute of Technology and local listings. In line with the ethics approval, only right-handed people between the ages of 18 and 65 were recruited.

All participants had normal or corrected-to-normal vision and had not experienced VR with either vibrotactile feedback at the fingertip or any form of force feedback, including EMS. Participants were informed about the nature of the experiment, recording and anonymization procedures. Each subject signed a consent form. Participants were compensated 10 euros or 1 study participation hour (course credit) per hour.

Before further analysis, data from the first subject were removed due to data recording errors.

### 2.2 Apparatus

A virtual environment was designed in Unity3D (Unity Software Inc., San Francisco, CA, USA) and presented through the HTC Vive Pro (High Tech Computer Co., Taoyuan, Taiwan) featuring a 1, 440 × 1, 600 per-eye resolution and a 98° horizontal field of view (for technical details, see: https://vr-compare.com/headset/htcvivepro). An HTC VIVE tracker (High Tech Computer Co., Taoyuan, Taiwan) was used to capture the position of the hand (for technical details, see: https://vr-compare.com/accessory/htcvivetracker3.0).

One vibrotactile actuator (Model 308–100 from Precision Microdrives, London, UK) worn on the fingertip was used to generate (vibro)tactile feedback, with 0.8 g at 200 Hz. This motor measures 8 mm in diameter, making it ideal for the fingertip. The vibration feedback was driven at 70 mA by a 2N7000 Metal Oxide Semiconductor Field-Effect Transistors (MOSFET), which was connected to an Arduino output pin at 3 V. To generate force feedback, we actuated the index finger via EMS, which was delivered via two electrodes attached to the participants' extensor digitorum muscle. We used a medically compliant EMS device (Rehastim, Hasomed, Germany), which provides a maximum of 100 mA and is controllable via USB. The EMS was pre-calibrated per participant to ensure pain-free stimulation and robust actuation.

**Figure 1:**
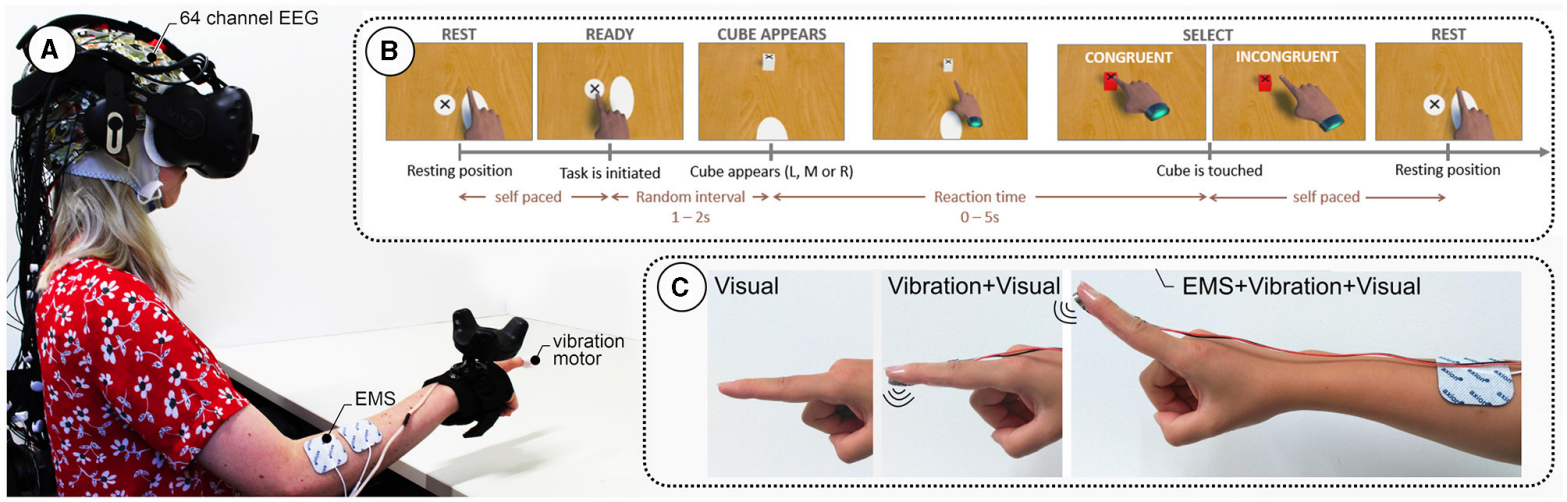
**(A)** Experimental setup showing a participant wearing a 64-channel electroencephalography (EEG) cap and a virtual reality (VR) headset. The participant's right arm is equipped with electrical muscle stimulation (EMS) electrodes and a vibration motor under the index finger. **(B)** Task sequence: The participant starts in a resting position and initiates the task at their own pace. After a random interval of 12 s, a cube appears in one of three positions (Left, Middle, Right). The participant reaches for the cube, with the selection being either congruent or incongruent. The task ends when the cube is touched, followed by a return to the resting position. **(C)** Different feedback modalities used in the study: Visual feedback only, combined Vibration and Visual feedback, and EMS combined with Vibration and Visual feedback.

EEG data were recorded from 64 actively amplified electrodes using BrainAmp DC amplifiers from BrainProducts (BrainProducts GmbH, Gilching, Germany). Electrodes were placed according to the 10–20 system (Homan, 1988). Custom EEG cap spacers^1^ were used to ensure a good fit and less discomfort due to the VR–EEG combination. After fitting the cap, all electrodes were filled with conductive gel to ensure proper conductivity. Electrode impedance was brought below 5K Ohm when possible. See Figure 1A for the full experimental setup.

### 2.3 Experimental design

The data were collected during a repeated reach-to-tap task in a 2 × 3 study design with the within-subject factors feedback congruity and modality.

#### 2.3.1 Task

Participants performed the task sitting in front of a table, virtually as well as physically. The interaction flow, depicted in Figure 1B, was as follows: participants moved their hands from the resting position to the ready position to indicate they were ready to start the next trial. Participants waited for a new target (a cube) to appear in one of three possible positions (center, left, and right), all located at the same distance from the ready position button on the table. The time for a new target spawning was randomized between 1 and 2 s. A black cross on the top of the cube indicated the location participants were instructed to tap. Then, participants completed the task by tapping the target with their index finger. Tapping success was indicated through three different sensory modalities (see Figure 1C):

##### 2.3.1.1 Visual-only feedback (visual)

Touching the virtual cube led to a change in its color from white to red. No haptic feedback was given.

##### 2.3.1.2 Tactile feedback (vibro)

In addition to visual feedback, touching of the virtual cube was additionally confirmed by a 100 ms vibrotactile stimulus.

##### 2.3.1.3 Force feedback (EMS)

In addition to visual and tactile feedback, participants received 100 ms of EMS via two electrodes at the extensor digitorum muscle.

After a target was tapped, participants moved back to the resting position. Here, they could rest before starting the next trial. To maximize EEG data quality, participants were instructed to remain in a calm upright seated position while carrying out the reaching movement. Furthermore, they were instructed to be precise and keep a good pace. However, no feedback was given on the accuracy and speed of their task completion.

#### 2.3.2 Feedback congruity/visuo-haptic mismatches

The *key* experimental manipulation in these data is the introduction of prediction errors occurring at different levels of immersion rendered through the haptic modalities. Therefore, to allow assessment of the effects of flawed sensory feedback, the feedback congruity was manipulated in a subset of the trials; see Figure 1B.

##### 2.3.2.1 Match trials (C), 75% of the trials

Feedback stimuli were presented upon tapping the object exactly when participants expected them to occur based on the available visual information (finger touching the target in the virtual environment).

##### 2.3.2.2 Mismatch trials (M), 25% of the trials

Feedback stimuli were triggered prematurely. Specifically, we introduced a temporal delta between the expected time of feedback, based on proprioceptive and visual information (finger touching the target in the virtual environment), and the actual time of feedback. This delta was realized by changing the cue triggering the hit sphere (sphere collider) around the virtual cube. While using a collision detection volume of the exact size of the cube in the match trials, we enlarged the radius of a cue-triggering sphere by 350% in the mismatch trials. This decision was based on the study design by Singh et al. (2018), in which they showed that VR users can detect a visual mismatch at ~200% of visual offset from the target. Based on pilot tests, we decided to extend the offset to 350% to increase the salience of the mismatch. One alternative solution to this, would be to alter the control-to-display ratio (Terfurth et al., 2024). This allows for a more precise timing of the violation with respect to the ballistic and corrective phases of the motion.

#### 2.3.3 Procedure

The experiment consisted of five phases that started with (1) a setup phase, (2) a calibration phase, and (3) a short training phase. For training purposes, we asked participants to wear the VR headset for a maximum of 24 practice trials. Overall, the EEG fitting, calibration, and practice trials took ~30 min (with two experimenters). In step (4), the task itself, the procedure varied between participants.

Per participant, 300 trials were recorded for the Visual and Vibro feedback condition. For the EMS condition, 100 trials were recorded, as this condition was exploratory, and we did not want to put too much strain on participants with a feedback channel that not many people are familiar with.

The order of the Visual and Vibro conditions was counterbalanced across participants, with the EMS condition always being the last block. EMS trials were only collected for 11 participants. The EMS condition was added as an exploratory part of the study, and 11 participants were deemed enough for exploratory analyzes. The EMS condition was always presented as the last block in order to prevent overshadowing of the strong stimuli of the EMS simulations on the other conditions. Because of this positioning, no impact on the visual and visuo-tactile contrast was implied. The general blocked design of the interface conditions was chosen to emphasize the influence of the additional haptic channels while attenuating higher order interactions, such as a prediction error about the upcoming interface condition.

At the end of each condition, we presented four questions from the standard igroup presence questionnaire (IPQ) (Schubert, 2003), in particular: The general presence item (G1), The second item of the realness subscale (REAL2), the fourth item of the spatial presence subscale, and the first item of the involvement subscale (INV1). The questionnaire was implemented into the virtual environment.

### 2.4 Data records

EEG, motion capture, and an experiment marker stream were recorded and synchronized using “load_xdf” from labstreaminglayer (https://github.com/sccn/labstreaminglayer). The XDF files were then converted to BIDS format (Gorgolewski et al., 2016; Pernet et al., 2019; Jeung et al., 2023) and the data are available online (https://openneuro.org/datasets/ds003846/versions/2.0.2). Motion data of a head and hand rigid body conform to the BIDS-Motion specification (Jeung et al., 2023) as of March 26, 2024. EEG data were recorded with a sampling rate of 500 Hz and FCz as the reference electrode. Hand and head movements were sampled at 90 Hz when coming out of the HTC VIVE processing cascade.

A full repository including links to the data, experimental VR protocol (Unity), and publication resources can be found at: https://osf.io/x7hnm/.

## 3 Validation

We provide the code to fully reproduce our results (https://github.com/lukasgehrke/2021-Scientific-Data-Prediction-Error), starting with the conversion of the raw .xdf files to the BIDS format. To ensure the quality of the data set, event-related potential (ERP) and event-related spectral perturbation (ERSP) are reported here at moments of prediction violation.

### 3.1 Signal processing

Our pipeline uses parts of the BeMoBIL pipeline, which wraps and extends EEGLAB toolboxes (Delorme and Makeig, 2004; Klug et al., 2022). Statistical tests were then computed using MNE–Python (Gramfort et al., 2013).

#### 3.1.1 EEG

After removing non-experiment segments at the beginning and end of the recording, EEG data were resampled to 250 Hz. Next, bad channels were detected using the “FindNoisyChannel” function, which selects bad channels by amplitude, the signal-to-noise ratio, and correlation with other channels (Bigdely-Shamlo et al., 2015). Rejected channels were then interpolated while ignoring the electrooculogram (EOG) channel and finally re-referenced to the average of all channels, including the original reference channel FCz. After applying a high-pass filter at 1.5 Hz, time-domain cleaning and outlier removal were performed using adaptive mixture of independent component analyzers (AMICA) auto rejection (Palmer et al., 2011). Eye artifacts were removed using the ICLabel toolbox applied to the results from an AMICA (Pion-Tonachini et al., 2019). For this, the popularity classifier was used, meaning that all components having the highest probability for the eye class were projected out of the sensor data.

#### 3.1.2 Motion

Motion capture data were filtered with a 6 Hz low-pass filter and resampled to match the EEG sample rate. The first and second derivative were taken and subsequently filtered using an 18 Hz low-pass filter.

### 3.2 Detecting the time of movement onset and peak velocity

We obtained the time of movement onset and subsequent peak velocity by applying a velocity-based algorithm on the hand-motion time series. The algorithm used a simple two-step threshold approach to obtain a robust movement onset of the outward reaching motion. First, a robust onset was defined by the time point where the velocity first exceeded 50% of the maximum velocity between the trial start event and the successful object tap event. Next, a precise motion onset was defined by the first time point where the signal preceding the robust onset fell below 10% of the robust threshold value.

Subsequently, the peak velocity of the outward motion was determined by peak extraction using the MATLAB function “findpeaks” in the time window between the motion onset and the object tap event; see Knill and Pouget (2004).

### 3.3 Event-related brain activity and hand movement characteristics

Event-related time courses from both, band-pass filtered (0.1–15 Hz) electrode FCz (ERP), as well as the hand velocity (ERV) were extracted. ERPs were obtained from –100 to 600 ms around the “tap” event. ERVs were obtained from –500 to 500 ms around the maximum velocity peak; see Knill and Pouget (2004).

#### 3.3.1 Event-related spectral perturbations

Event-related spectral perturbations (ERSP) were obtained by extracting epochs from the trial onset, that is, spawn of the sphere, to the object tap. A pre-stimulus interval was included for later baseline correction. A spectrogram of all single trials was computed using the EEGLAB's “newtimef” function (3–100 Hz in logarithmic scale, using a wavelet transformation with three cycles for the lowest frequency and a linear increase with frequency of 0.5 cycles). The resulting spectograms were linearly time-warped to the movement onset and time of peak velocity.

### 3.4 Statistics

ERPs were baseline-corrected by subtracting the average amplitude of the last 100 ms preceding the trial start. To ascertain effects of both ERP and ERV, the linear mixed-effects model “sample condition + modality + 1|participantID” was fit at each time point. Effects were assessed using likelihood ratio tests for the main effects with Benjamini–Hochberg *p*-value correction for false discovery rate (Benjamini and Hochberg, 1995).

For ERSP, a spatiotemporal cluster test was conducted in comparison to power values in a –300 to –100 ms pretrial baseline window. The test was conducted for both contrasts: one test against a pre-stimulus baseline and one between conditions.

### 3.5 Results

**Figure 2:**
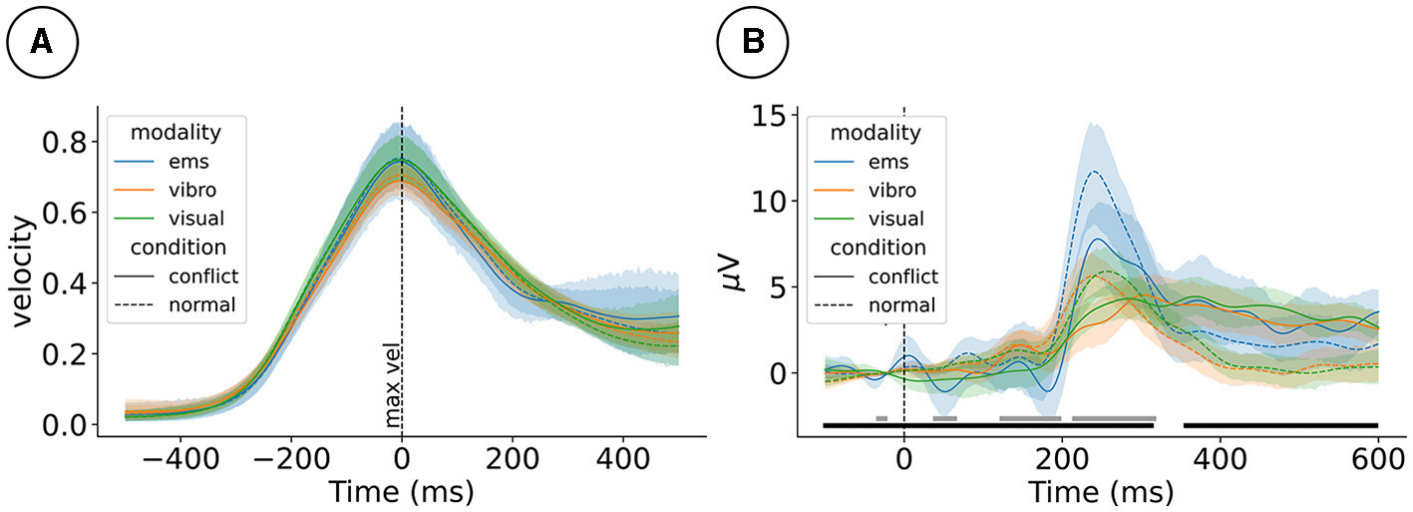
**(A)** Event-related hand velocity (ERV) and **(B)** event-related potentials (ERP) at electrode FCz for the 2 (mismatch condition) × 3 (haptic modality) design. ERV is plotted from –500 to 500 ms around the peak velocity. ERP is plotted from –100 to 600 ms around the tap event. Gray (haptic modality) and black (mismatch condition) blocks at the bottom mark effects. ems, electrical muscle stimulation.

We observed similar motion profiles across the three different haptic modalities. The oddball-like mismatch manipulation did not change how participants moved. This can be seen in Figure 2A, which shows the hand velocity time-locked to the velocity peak of the motion.

Visuo-haptic mismatches impacted event-related processing as picked up by the EEG. As reported in our previous studies, we observed that mismatch stimuli impacted the ERP, for example, at electrode FCz 170 ms post-stimulus, $\chi_{\left(1\right)}^{2}=25.7,p<0.0001$; see Figure 2B. Furthermore, the level of haptic immersion impacted the ERP, for example, electrode at 170ms post-stimulus ($\chi_{\left(1\right)}^{2}=16.7,p=0.0002$).

**Figure 3:**
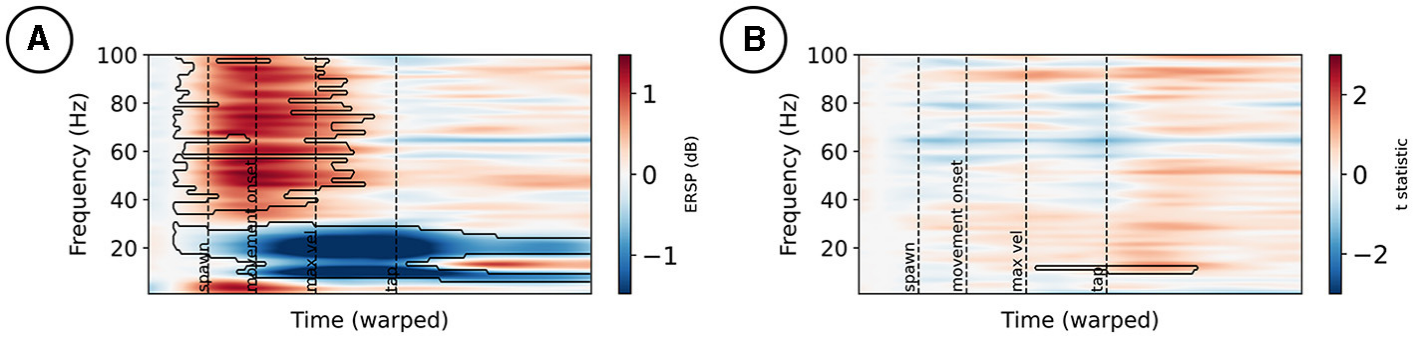
Event-related spectral perturbations at electrode FCz **(A)**. Changes from a –300 to –100 ms pre-stimulus baseline are marked by a black contour. **(B)** *t*-statistic of the mismatch condition contrast, with effects marked by a black contour.

For simplicity, only the test against baseline and the main effect of the mismatch condition is plotted for electrode FCz in Figures 3A, B, respectively. At FCz, ERSPs appear in high frequencies early on during the movement, with a positive change compared to baseline. Furthermore, a negative change compared to baseline power appears in the alpha and beta frequency ranges during the movement, with a peak following maximum velocity; see Figure 3A. Mismatch stimuli affected the spectral power at FCz in lower frequency bands around the tap event; see Figure 3B.

## Data availability statement

Publicly available datasets were analyzed in this study. This data can be found at: https://openneuro.org/datasets/ds003846.

## Ethics statement

The studies involving humans were approved by Ethik-Kommision (EK), Institut für Psychologie und Arbeitswissenschaft (IPA), TU Berlin. The studies were conducted in accordance with the local legislation and institutional requirements. The participants provided their written informed consent to participate in this study.

## Author contributions

LG: Writing – review & editing, Writing - original draft, Visualization, Validation, Supervision, Software, Project administration, Methodology, Investigation, Formal analysis, Data curation, Conceptualization. LT: Writing – review & editing, Writing – original draft. SA: Writing – original draft, Methodology, Investigation, Data curation, Conceptualization. KG: Writing – review & editing, Resources, Project administration, Funding acquisition.

## Footnotes

^1^ https://grabcad.com/library/adapter-for-vr-eeg-setups-1
